# Sanguinarine modulate gut microbiome and intestinal morphology to enhance growth performance in broilers

**DOI:** 10.1371/journal.pone.0234920

**Published:** 2020-06-19

**Authors:** Zhu-Ying Liu, Xiao-Long Wang, Shu-Qi Ou, De-Xing Hou, Jian-Hua He

**Affiliations:** 1 College of Animal Science and Technology, Hunan Agricultural University, Changsha, Hunan Province, China; 2 The United Graduate School of Agricultural Science, Faculty of Agriculture, Kagoshima University, Kagoshima, Japan; USDA-Agricultural Research Service, UNITED STATES

## Abstract

Sanguinarine is a bioactive compound as a quaternary benzophenanthridine alkaloid from plant of the *Macleaya cordata*, *Papaveraceae* family. The present study was conducted to investigate the effects of dietary sanguinarine supplementation on growth performance, serum biochemistry parameters, intestinal mucosal morphology and gut microbiome in yellow feathered broilers. Two hundred and seventy 1-d-old female broilers were randomly assigned to 3 treatments ① Basal diet (NG); ② Basal diet containing bacitracin methylene disalicylate (50mg/Kg diet) (ANT); ③ Basal diet containing sanguinarine (0.7 mg/ kg of feed) (SAG). The statistical results showed that dietary sanguinarine supplementation enhanced growth performance and decreased glucose, uric acid as well as urea nitrogen levels of broilers at 28d of age (P<0.05). The 16S rRNA gene sequence analysis revealed that sanguinarine significantly decreased the species from the phyla Bacteroidetes, and increased the species from phyla Firmicutes. Moreover, dietary sanguinarine supplementation improved mucosal morphology to achieve higher ratio of intestinal villus height to crypt depth (P < 0.05), and decreased the concentrations of TNF-α and IL-4 in jejunum mucosal. This study demonstrated that sanguinarine supplementation in the diet of yellow feathered broilers improved intestinal morphology and microbiota community structure to promote growth performance on 1-28d.

## Introduction

Sanguinarine (C_20_H_14_NO_4_) is a quaternary benzophenanthridine alkaloid from phenylalanine in plants of the *Macleaya cordata*, *Papaveraceae* family[1]. *Macleaya cordata* was recognized by the European Food Safety Authority as a feed additive for animal production[2]. Sangrovit^®^ is a commercial product extracted from *Macleaya cordata* which is composed of mainly sanguinarine and chelerythrine, and it is standardized to 1.5% sanguinarine[3]. Previous studies reported that sanguinarine displayed a wide range of pharmacological activities, such as antifungal, anti-inflammatory, antimicrobial, analgesic and anti-cancer properties[4–6]. Sanguinarine have been reported to cause toxicity in different living system. Sanguinarine is a toxin that kills animal cells through its action on the Na+-K+-ATPase transmembrane protein[7]. Sanguinarine also displayed significant cytotoxicity on different types of cancer cells[8–10]. The acute oral LD50 in rats were reported to be about 1,658 mg/kg of sanguinarine [11]. Despite the toxicity and mutagenicity of sanguinarine, the compound is still extensively studied due to the possibility of synthesis of derivatives with reduced toxicity, an average daily oral dose of alkaloids up to 5 mg per 1 kg animal body weight on pigs proved to be safe [12]. Sanguinarine might be metabolized to nontoxic dihydrosanguinarine in intestine after oral administration in pigs by intestinal mucosa microsomes, cytosol, and flora[13]. Dietary supplementation with sanguinarine has shown beneficial effects in enhancing the growth performance of pigs[14–16], broiler chickens[17,18], cattle[19,20], and fish[21,22]. A recent study has shown that dietary supplementation of sanguinarine enhanced growth performance and the relative length of intestine, and altered gut microbiota in broiler chickens[23]. However, those previous studies utilized a normal colony-counting method to analyze intestinal microflora with no information on microbial diversity. The microbiome of the broiler chicken gastrointestinal tract is essential for the gut homeostasis and the host metabolism. They play an important role in maintaining the health of the host, such as the positive impact on the immune system and productivity[24,25]. Interestingly, the composition of broiler chicken gut microbiota has recently been reported to be related to an average daily weight gain and body weight[26,27], feed efficiency[28], feed conversion and feed intake[29]. Moreover, probiotics, prebiotics, and phytobiotics have been used to positively regulate the microbial community in the gastrointestinal tract of chicken[22,30].

Antibiotics are the main additives used in the poultry feed to improve growth, however, restrictions on the use of antibiotics imposed by China from July 2020. Therefore, alternatives have been actively investigated to replace the usual growth promoting agents. Our preliminary data showed that sanguinarine supplemented at the rate of 0.7 mg/kg diet increased lymphocyte proliferation and improved immunity in yellow broiler chickens (unpublished results). Sanguinarine is also reported to have an effect on intestinal health, including intestinal morphology, microbiota and metabolic profiles in broiler chickens[26,31]. However, only a few studies have analyzed the gut microbiota profiles in detail when broiler chicken was supplemented with sanguinarine. The yellow feathered broiler is a local breed raised for meat in China[32], with a lower adipogenic trend and higher meat efficiency than those of normal commercial breeds.

Therefore, this study was conducted in order to evaluate the effect of using growth promoters (antibiotics and sanguinarine) in diets on growth performance, serum biochemistry parameters and gut microbiome of broilers, then to explore the relationship between these growth performance and gut microbiome by clarifying the changes in gut microbiota and intestinal morphology with 16S rRNA gene sequencing and hematoxylin staining when yellow feathered broiler was supplemented with sanguinarine at a dietary level of 0.7mg/kg.

## Methods

### Ethics approval

All the experimental procedures were approved by the Institutional Animal Care and Use Committee of Hunan Agricultural University.

### Animals, diets, and experimental design

A total of two hundred and seventy (270) one day old female yellow-feathered broiler chickens (average BW = 30.3g) were obtained from Hunan Wenshi Poultry Industrial Development Company, China. Healthy chicks were randomly assigned to 3 treatments with 6 replicates (15 chickens of one cage as a replicate). The birds were treated for 56 days with following diets ① Basal diet (NG); ② Basal diet containing bacitracin methylene disalicylate (BMD, 50mg/Kg diet) (ANT); ③ Basal diet containing sanguinarine (0.7 mg/ kg of feed) (SAG).

Each cage had raised wire floors and contained a self-feeder and waterer. The room temperatures were adjusted to 32°C in the first week, 30°C in the second week, 28°C in the third week, and then 25°C to the end. Traditionally, yellow broiler chickens were always divided into an early phase (1-28day) and late phase (29-56day). The diets used in this study were formulated based on the nutrient requirements of yellow-feathered broiler chickens (China, NY/T 33–2014) and the NRC (1994) (Table 1). All birds were vaccinated against coccidiosis at placement.

**Table 1:** The composition of the basal diet.

Item	Starter(1-28day)	Finisher(29-56day)
Ingredient, %		
Ground yellow maize	56.65	58.55
Soybean meal	36	33.5
Soybean oil	3.0	3.5
Di-calcium phosphate	1.8	1.9
Limestone	1.0	1.0
NaCl	0.3	0.3
DL-Met	0.1	0.1
Choline chloride	0.15	0.15
Premix^1^	1.0	1.0
Nutrient level^2^		
ME,MJ/kg	12.22	12.43
CP, %	20.10	19.2
Lys, %	1.02	0.96
Met, %	0.42	0.41
Cys,%	0.32	0.31
Ca,%	1.11	1.13
Available P, %	0.54	0.55

DL-Met, DL-Methionine; ME, Metabolizable Energy; CP, Crude Protein.

^1^Supplied, per kilogram of diet: Cu, 10 mg; Fe, 90 mg; Mn, 90 mg; Zn, 50 mg; Se, 0.2 mg; I, 0.4 mg; Co, 0.4 mg; vitamin A, 5,000 IU; cholecalciferol, 500 IU; vitamin E, 10 IU; riboflavin, 6.0 mg; pantothenic acid, 12 mg; niacin, 35 mg; cobalamin, 10 μg; biotin, 0.8 mg; folic acid,0.8 mg; thiamine, 1.5 mg; and pyridoxine, 1.5 mg.

^2^ Based on the Nutrient Requirements of yellow broilers (China, NY/T 33–2014) and the Nutrient Requirements of Broilers (NRC,1994).

### Preparation of sanguinarine

Sanguinarine was extracted from *Macleaya cordata* at the National and Local Union Engineering Research Center of Veterinary Herbal Medicine Resource and Initiative, Hunan Agricultural University with ethanol as described previously[33,34]. Extracted sanguinarine was separated with silica gel column chromatography and the purity of sanguinarine was measured to be 99% by HPLC.

### Sample collection and preparation

In a 56-day experiment, individual body weight of the birds was measured at 28 and 56 days old. Similarly, feed intake per cage was recorded daily. The growth performance of broilers was calculated by the average daily feed intake (ADFI), average daily body weight gain (ADG) and feed/gain ratio (FCR) were calculated according to the data from each cage.

On days 28 and 56, six birds were randomly selected from each group (1 bird from each cage) and were fasted for 12 hours before been sacrificed. Immediately, before exsanguination, 5 mL of blood sample per bird was collected by venipuncture of the wing vein. Serum was obtained by centrifugation at 3000×*g* for 10 min at 4°C and stored at -20°C until serum biochemical analyses.

At the same time, after the birds have been euthanized, approximately 0.5 g of fecal samples was immediately taken from the middle of the cecum. Feces collected from 2 birds from each group were pooled to create one composite sample. The composite cecal samples were collected into 1.5 mL Eppendorf tubes and quickly frozen in liquid nitrogen before being transported to the laboratory and stored at -80°C until the 16S rRNA gene sequence analysis of cecal microbiota.

Thereafter, the small intestine was promptly moved out and divided into 3 parts: Duodenum (from the pylorus to the distal point of entry of the bile ducts), jejunum (Meckel’s diverticulum marked the end point of the jejunum), and ileum (the ileocecal junction marked the end of the ileum). One cm segment of the intestine was cut from the midpoint of each of the duodenum, jejunum, and ileum. These intestinal tissue samples were lightly flushed with physiological saline (154 mmol/L), blotted dry with filter paper and fixed into 4% paraformaldehyde fix solution at 4°C until further analysis of intestinal mucosal morphology, which was completed within 2 weeks after sample collection. Jejunal segments (10cm in length) were opened longitudinally and the contents were flushed with ice-cold PBS. Mucosa was collected by scraping using a sterile glass microscope slide at 4°C, rapidly frozen in liquid N2 and stored at −80°C for the analysis.

### Serum biochemical parameters

Serum total protein (TP), glucose(GLU), albumin(ALB), globulin(GLB); triglycerides (TG), total cholesterol (TC), high density lipoprotein (HDL), low density lipoprotein (LDL), alanine aminotransferase (ALT), aspartate aminotransferase (AST), blood urea nitrogen (BUN) and uric acid (UA) were analyzed using BS-200 automatic blood biochemical analyzer (Mindray, Shengzhen, China).

### Mucosal cytokines

Jejunal mucosa (0.5 g per sample) was weighted, diluted with 9 mL of 0.9% saline and homogenized at 4°C, and then centrifuged at 6,000 × g for 15 min at 4°C. The supernatant was harvested into 1.5 mL Eppendorf tubes. The concentrations of tumor necrosis factor-a (TNF-α) and IL-4 were analyzed by ELISA (Lan Bao Biological Technology Co. Ltd., Hangzhou, China).

### DNA extraction and cecal microbiota analysis of fecal samples

The DNA was extracted from fecal samples (three composite cecal samples from each group) using a Stool DNA Isolation Kit (Tiangen Biotech Co., Ltd., Beijing, China). The V3-V4 hypervariable region of the bacterial 16S rRNA gene was amplified by PCR with the forward primer 338F: 5′- ACTCCTACGGGAGGCAGCAG-3′ and the reverse primer 806R: 5′-GGAC- TACHVGGGTWTCTAAT-3′. For each cecal sample, 10-digit barcode sequence was added to the 5’ end of the forward and reverse primers (provided by Allwegene Company, Beijing). The component and volume of the reagents in the PCR amplification system concluded DNA sample 5 μL (30ng); Forward Primer (10uM), 2 μL; Reverse Primer (10uM) 2 μL; dNTPs (2.5mM) 4μ L; 10 * Pyrobest Buffer 5μL; Pyrobest DNA Polymerase 3 μL; ddH2O 31.7 μL (Total 50μL). Cycling parameters were 98°C for 1 min, followed by 30 cycles at 98 °C for 10 s, 57 °C for 30 s, and 72 °C for 30 s, and a final extension at 72 °C for 10 min. PCR products were mixed in equidensity ratios and purified with GeneJET Gel Extraction Kit (Thermo Fisher Scientific Inc., Schwerte, Germany), quantified using Real-Time PCR, and sequenced at Allwegene Company, Beijing. Two hypervariable regions of 16S rRNA, the V3 and V4 region were used to identify the vast majority of bacteria based on 16S rRNA sequencing. The sequences were clustered into operational taxonomic units (OTUs) at a similarity level of 97%, to generate rarefaction curves and to calculate the richness and diversity indices. OTUs representing <0.005% of the population were removed and taxonomy was assigned by the Ribosomal Database Project (RDP) classifier. Sequence data analysis was mainly performed using QIIME (v1.8.0, University of Colorado, Denver, CO, USA) and R packages (v3.2.0, Bell Labs Technology Showcase, Murray Hill, NJ, USA). The alpha diversity indices, Chao1 richness estimator, and the Shannon diversity index were calculated using the OTU table in QIIME[35]. Beta diversity analyses was performed using UniFrac distance metrics and visualized by principal coordinates analysis (PCoA) of the unweighted Unifrac distance matrices. The significance of microbiota structure differentiation among groups was assessed by PERMANOVA (permutational multivariate analysis of variance) and ANOSIM (analysis of similarities) using the R package “vegan”. Taxa abundances at different taxonomies were statistically compared among groups by Kruskal-Wallis and visualized as box plots.

### Measurement of intestinal mucosal morphology

One cm intestinal tissue samples of the duodenum, jejunum, and ileum were embedded in paraffin. A microtome (RM-2235, Leica Microsystems AG., Hessen, Germany) was used to make 5 or 6 μm slices that were mounted in glass slides and subsequently stained with hematoxylin and eosin (H&E staining). Villus height (from the tip of the villus to the villus-crypt junction) and crypt depth (from villus-crypt junction to the base of the crypt) were measured under an Olympus Van-Ox S microscope (Opelco, Washington, DC) using an image analysis software (Image-Pro, Media Cybernetics, Inc., Silver Springs, MD). Six sections were taken from each intestine, and the height of the ten largest villi and the deepest crypt depth were selected for each section. The villus height/crypt depth (V/C) value was calculated as described previously[36].

### Statistical analysis

Data were expressed as mean ± SEM and analyzed by one way ANOVA for single factor design using SPSS 20.0 (SPSS, Inc., Chicago, IL, USA). The mean differences among different groups were separated by Tukey’s tests. A level of *P* < 0.05 was used as the criterion for statistical significance. The statistical analyses used in the assessment of microbial community structure (16S rRNA sequencing) was described in Section 2.2.3.

## Results

### Growth performance

The growth performance of birds was shown in Table 2. Throughout the entire experimental period, birds in ANT group exhibited higher ADG when compared with birds fed on the NG group (p<0.001). SAG group had higher ADG than that in NG group at 28d (p<0.001), but not significantly different at 56d (p>0.05). SAG group had higher ADFI than NG group at 28d of age (p = 0.04), but no significant difference (p = 0.814) at 56d. Birds in ANT group and SAG groups had a lower FCR than NG group at 28d of age (p<0.001). However, the ANT group had the lowest FCR among the groups at 56d (p = 0.003).

**Table 2:** Effects of sanguinarine on growth performance in yellow-feathered broilers at 28 and 56 days of age.

Item	Time(d)	NG	ANT	SAG	SEM	P-value
BW(g)	28	615.26^b^	665.37^a^	661.53^a^	6.09	<0.001
	56	1849.57^b^	1968.12^a^	1877.77^b^	15.67	<0.001
ADG(g)	1–28	20.89^b^	22.68^a^	22.54^a^	0.22	<0.001
	1–56	32.49^b^	34.60^a^	32.99^b^	0.29	<0.001
ADFI(g)	1–28	44.91^b^	46.16^ab^	46.73^a^	0.33	0.04
	1–56	79.09	78.96	78.45	0.47	0.814
FCR(g:g)	1–28	2.15^a^	2.04^b^	2.07^b^	0.02	<0.001
	1–56	2.44^a^	2.28^b^	2.37^a^	0.02	0.003

NG: basal diet; ANT: basal diet group with 50mg/Kg BMD; SAG: sanguinarine supplemented with 0.7 mg/kg of diet; BW, body weight; ADG, average daily body weight gain; ADFI, average daily feed intake; FCR, feed/gain ratio.

^a,b^ within a row, values with different superscripts differ significantly (P < 0.05). Each mean represents 6 replicates.

### Serum biochemical parameters

**Fig 1:**
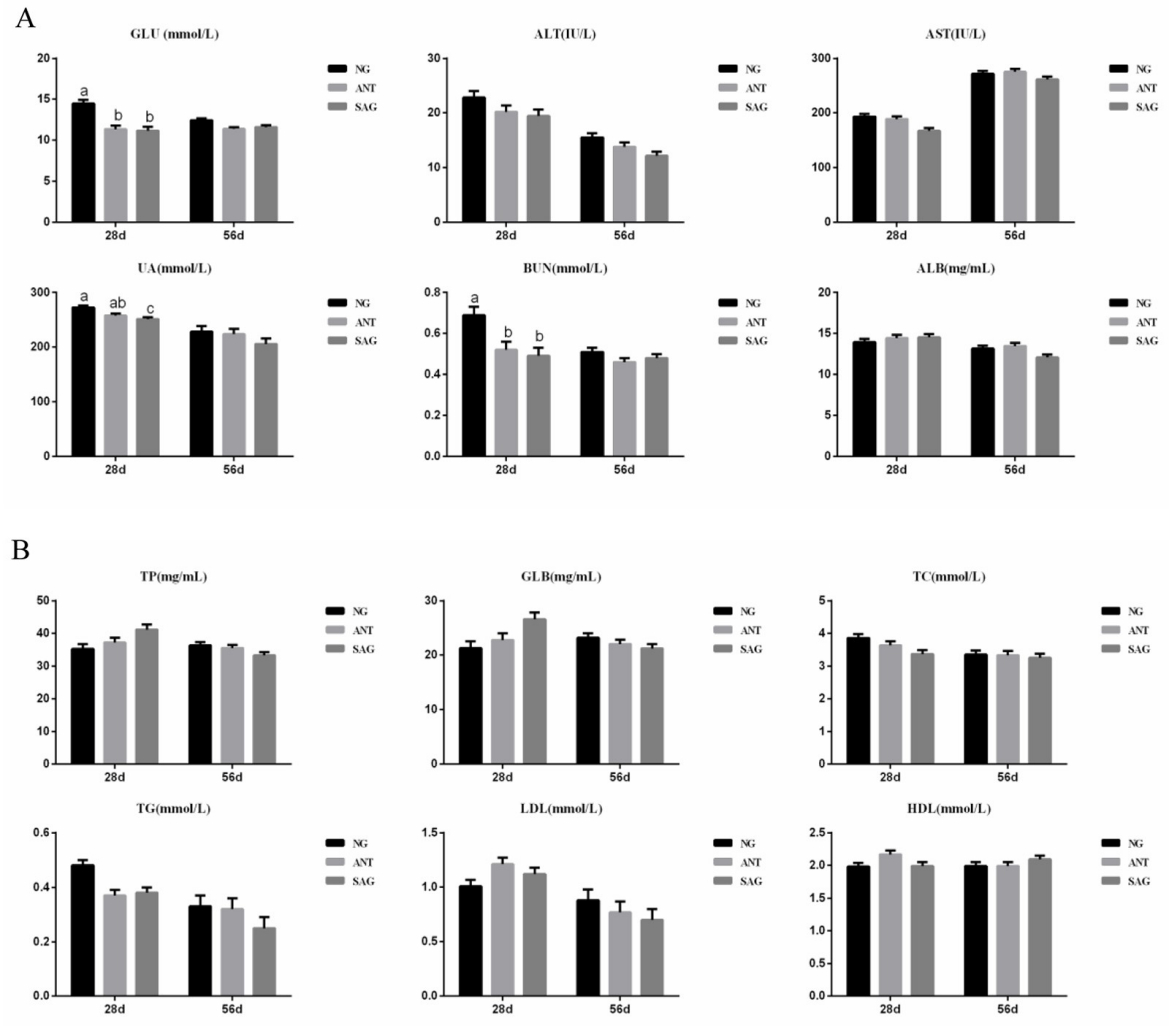
Effects of sanguinarine on serum biochemistry parameters in yellow-feathered broilers. (A) The concentration of GLU, UA, BUN, ALT, AST, and ALB in yellow broilers; (B) The concentration of TP, GLB, HDL, LDL,TC, and TG in yellow broilers; Different superscripts lowercase letters within each group mean different significantly (P<0.05). Each mean represents 6 replicates. NG: basal diet; SAG: sanguinarine supplemented with 0.7 mg/kg of diet; ANT: basal diet group with 50mg/Kg BMD; GLU: glucose; UA: uric acid; AST: aspartate aminotransferase; BUN: blood urea nitrogen; TG: triglycerides; ALT: alanine aminotransferase; TP: total protein; ALB: albumin; GLB: Globulin; TC: total cholesterol; LDL: low density lipoprotein; HDL: high density lipoprotein.

The results of serum biochemistry parameters are shown in Fig 1. Throughout the entire experimental period, the majority of parameters(TP, GLB, ALB, AST, ALT, HDL, LDL, TC, and TG) displayed no significant deviation between treated and control groups (p>0.05). At 28d, the concentration of GLU and BUN of broilers in the ANT and SAG groups was lower than the NG group, the concentration of UA of broilers in the SAG groups was lower than the NG group.

### Inflammatory response

**Fig 2:**
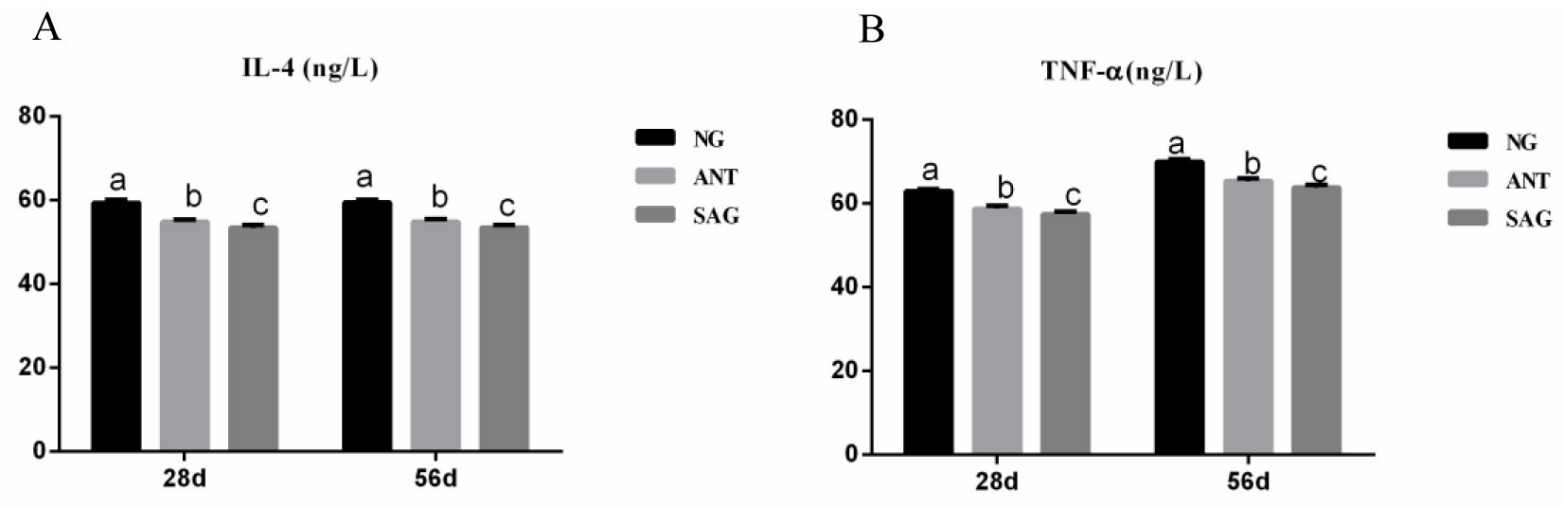
Effects of sanguinarine on mucosa inflammatory response in yellow-feathered broilers. (A) The concentration of IL-4 in jejunal mucosa; (B) The concentration of TNF-α in jejunal mucosa;a,b within a row, values with different superscripts differ significantly (P < 0.05). Each mean represents 6 replicates. NG: basal diet; SAG: sanguinarine supplemented with 0.7 mg/kg of diet; ANT: basal diet group with 50mg/Kg BMD; TNF-α: tumour necrosis factor α; IL-4: interleukin-4.

As shown in Fig 2, the ANT group and SAG group had a lower concentration of IL-4 and TNF-α in jejunal mucosa compared with NG group at 28d and 56d (P< 0.05).

### Diversity of the cecal microbiota of broilers

**Fig 3:**
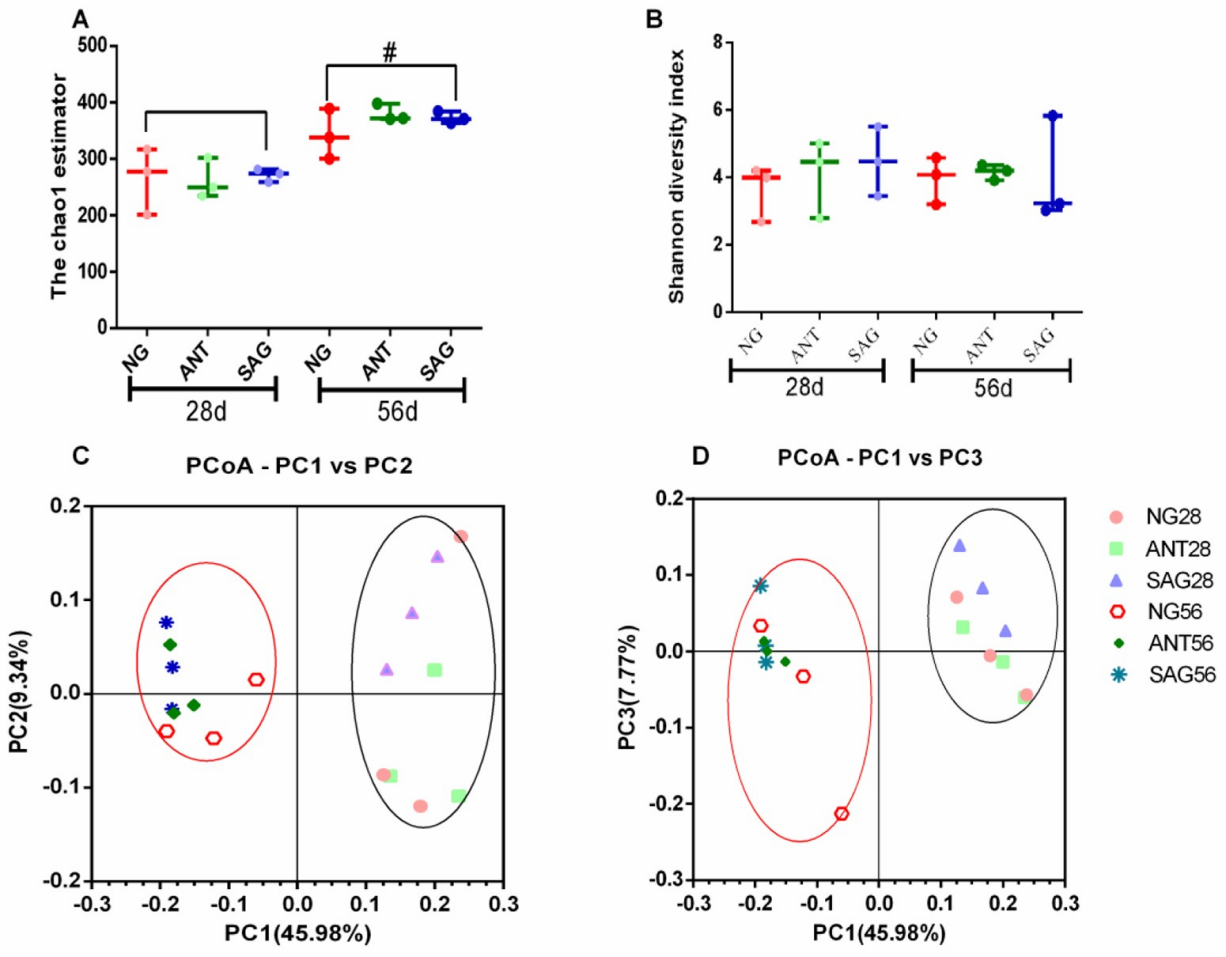
Effect of sanguinarine on bacterial communities’ diversity in cecal of yellow-feathered broilers. (A, B) Cecal taxa richness assessed by α-diversity analyses using chao1and Shannon method. (C,D) Cecal microbial community β-diversity (unweighted Unifrac, P>0.05), which was demonstrated using principal coordinates analysis (PCoA) of the unweighted Unifrac distance matrices. Microbial communities clustered according to the stage (28d vs 56d); however, there was no obvious clustering according to sanguinarine supplementation. Percent of dataset variability explained by each principal coordinate is shown in the axes titles. # represents p < 0.05 compared with the early stage (28d) and later stage (56d). Each dot represents one sample and each group is denoted by a different color and shape. NG: basal diet; SAG: sanguinarine supplemented with 0.7 mg/kg of diet; ANT: basal diet group with 50mg/Kg BMD.

To determine the effect of sanguinarine and BMD on the baseline microbial community structure, we sequenced 16S rRNA gene libraries prepared from total cecal DNA and performed diversity analyses. Following trimming, assembly, and quality filtering of 350,000 sequences in the cecal samples, each of 400 bp was subsampled from each 16S library and used to assess the microbial community structure. To determine if cecal taxa richness was altered by our treatment groups, we performed α-diversity analyses using the chao1 and Shannon method (Fig 3A and 3B) which showed that there was no significant difference in α-diversity during the feeding period (p>0.05), but there were significant differences between early age and old age at chao1 index (28d vs 56d) (p<0.05). This result indicates that microbial community diversity changes with age. Furthermore, microbial community diversity in the groups were different but not significantly [β-diversity (unweighted Unifrac); (p>0.05)], but there were significant differences between early age and old age (28d vs 56d) (p<0.05), and this was demonstrated using principal coordinates analysis (PCoA) of the unweighted Unifrac distance matrices (Fig 3C and 3D).

### Composition of the cecal microbiota of broilers

**Fig 4:**
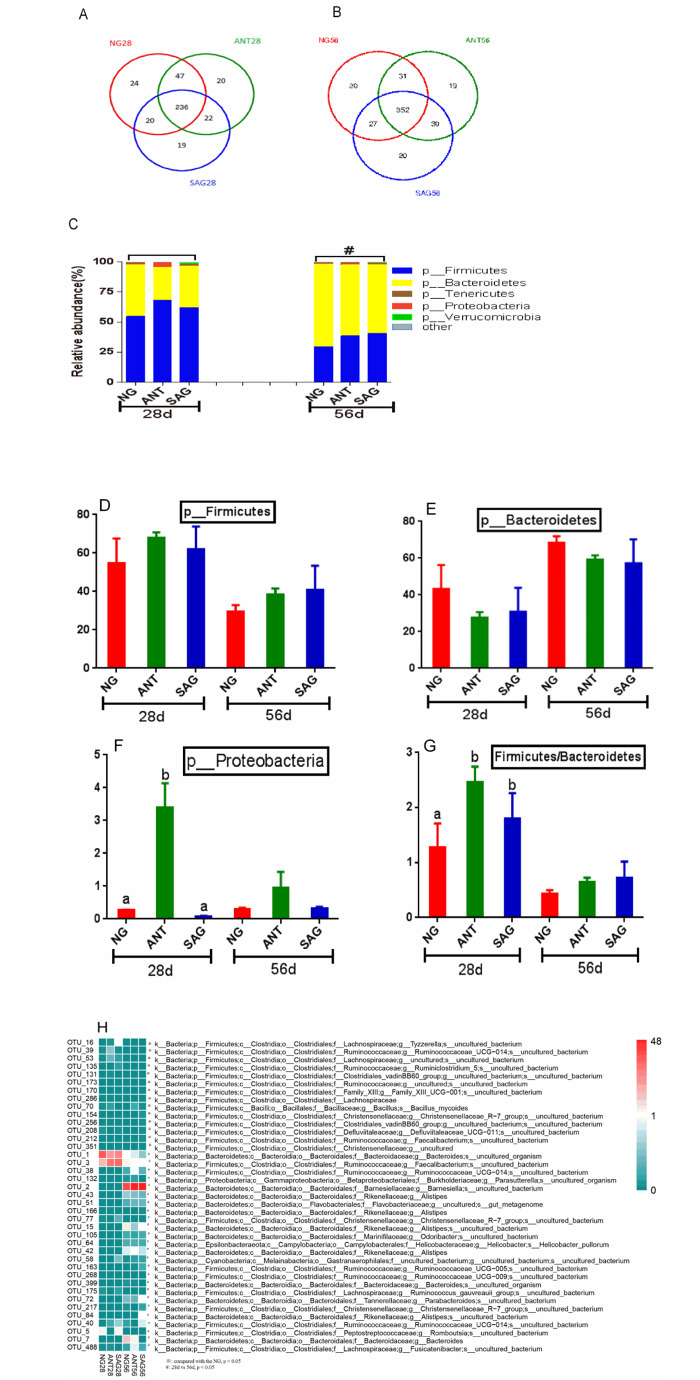
Effects of sanguinarine on cecal bacterial community in yellow-feathered broilers. The Venn diagram depicts OTUs that a shared or unique for different group(A, B); The composition and abundance distributions of each group at the phylum (C) and levels were shown using QIIME software Microbiota taxa composition at the phylum levels are shown(D, E, F, G). Heat map of 39 operational taxonomic units (OTUs) which were altered in abundance by sanguinarine and stage is shown (H), based on the redundancy analysis (RDA) model. OTUs with a relative abundance greater than 0.1% in at least in one group were selected and used to analyze these differences. The red and green colors indicate the relative abundances of OTUs that were more or less abundant. Different superscripts lowercase letters within each group mean different significantly (P<0.05).※,*represents p < 0.05 compared with the NG group in the same stage; # represents p < 0.05 compared with the early stage (28d) and later stage (56d). NG: basal diet; SAG: sanguinarine supplemented with 0.7 mg/kg of diet; ANT: basal diet group with 50mg/Kg BMD.

The microbiota taxa composition in the cecum of birds is shown at the phylum level in Fig 3. The three major bacteria phyla which were Firmicutes, Bacteroidetes, and Proteobacteria in the cecum, Verrucomicrobia and Tenericutes were also the main bacterial phyla in the cecum. As indicated, about 236 OTUs at 28d of age and 352 OTUs at 56d of age were shared among three groups of the bacterial community in cecum as shown in Venn diagram (Fig 4A and 4B). The NG group displayed the most unique OTUs among the three treatment groups but there was no significant difference. A significantly increased level of Proteobacteria at 28d age was observed in ANT group (p<0.05) (Fig 4F). The ratio of Firmicutes/ Bacteroidetes in ANT group and SAG group were higher than the NG group at 28d age (p<0.05) (Fig 4G). Next, we identified changes in the strain-specific key phenotypes which were affected by sanguinarine and BMD (Fig 4H). The results showed that the abundance levels of 14 OTUs were markedly changed by sanguinarine and BMD supplementation compared with NG group, while abundance levels of 25 OTUs were markedly changed by age (28d vs 56d). Particularly, among the 39 OTUs, 9 OTUs were significantly increased by sanguinarine. In addition, *Lachnospiraceae*, *Ruminococcaceae* and some genera within Firmicutes phylum were significantly more abundant in the groups offered sanguinarine supplemented diets.

### Relationships between the bacterial abundance and the metabolic parameters

**Fig 5:**
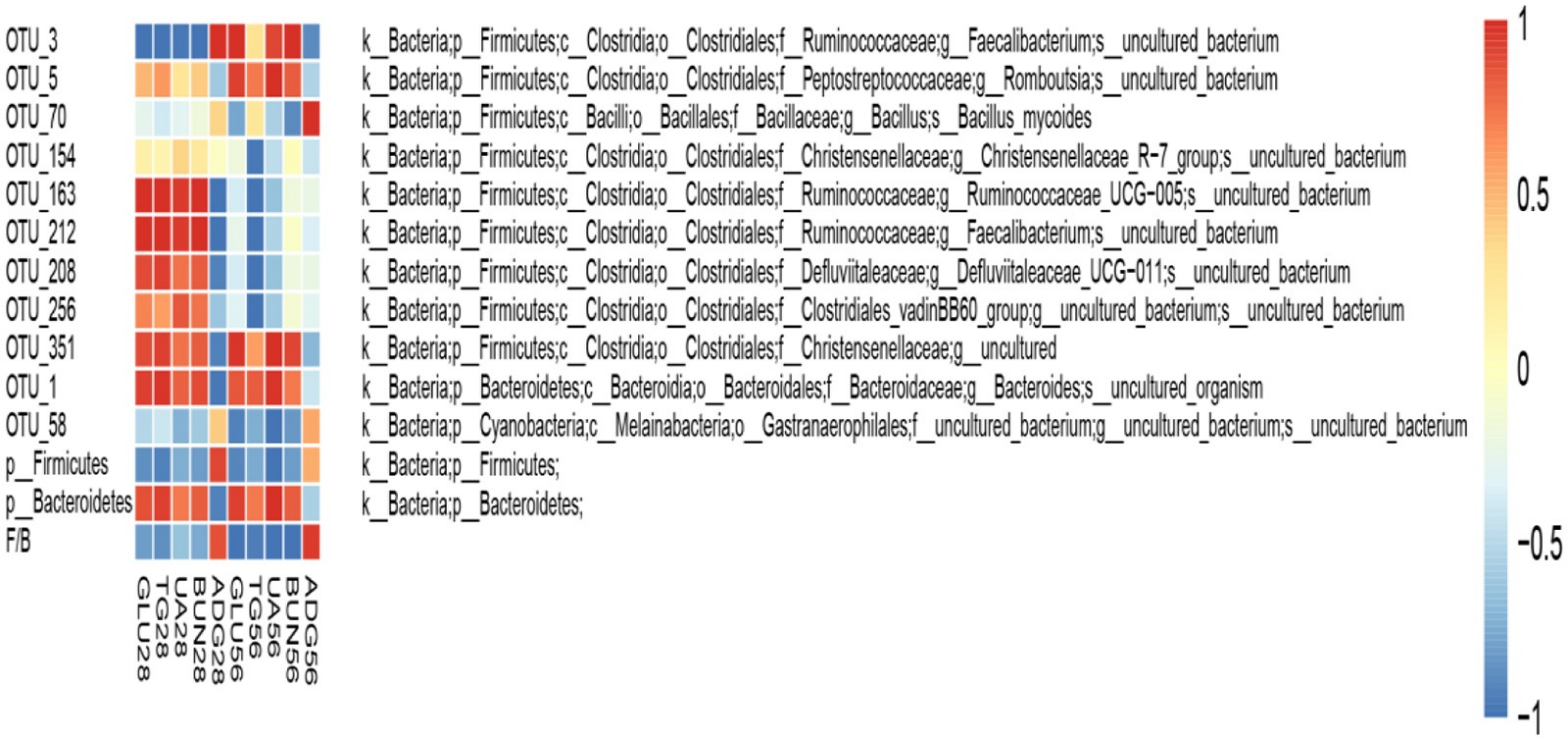
Relationships between the bacterial abundance and the metabolic parameters. Heat map of 11 OTUs and phylum, which were significantly associated with some metabolic parameters altered as determined by Spearman’s correlation coefficient. These 11OTUs were selected from the 39 OTUs which had significant changes between each group (in Fig 3). The red and blue colors indicate the correlation between the corresponding metabolic parameters and bacterial abundance. ADG: average daily body weight gain; BUN: blood urea nitrogen; UA: uric acid; TG: triglycerides; GLU: glucose; F/B: the ratio of Firmicutes/ Bacteroidetes.

To assess the relationships between OTUs and metabolic parameters altered by sanguinarine, Spearman’s correlation coefficient was employed. Among the 39 OTUs that were altered in abundance by sanguinarine and BMD as shown in Fig 3, 11 OTUs were markedly correlated with at least one of the following metabolic parameters: GLU, TG, UA, BUN, and ADG. Firmicutes, OTU_3 and the ratio of Firmicutes/ Bacteroidetes were positively correlated with the ADG; many OTUs were negatively correlated with ADG parameters (Fig 5).

### The change in intestinal mucosal morphology

**Fig 6:**
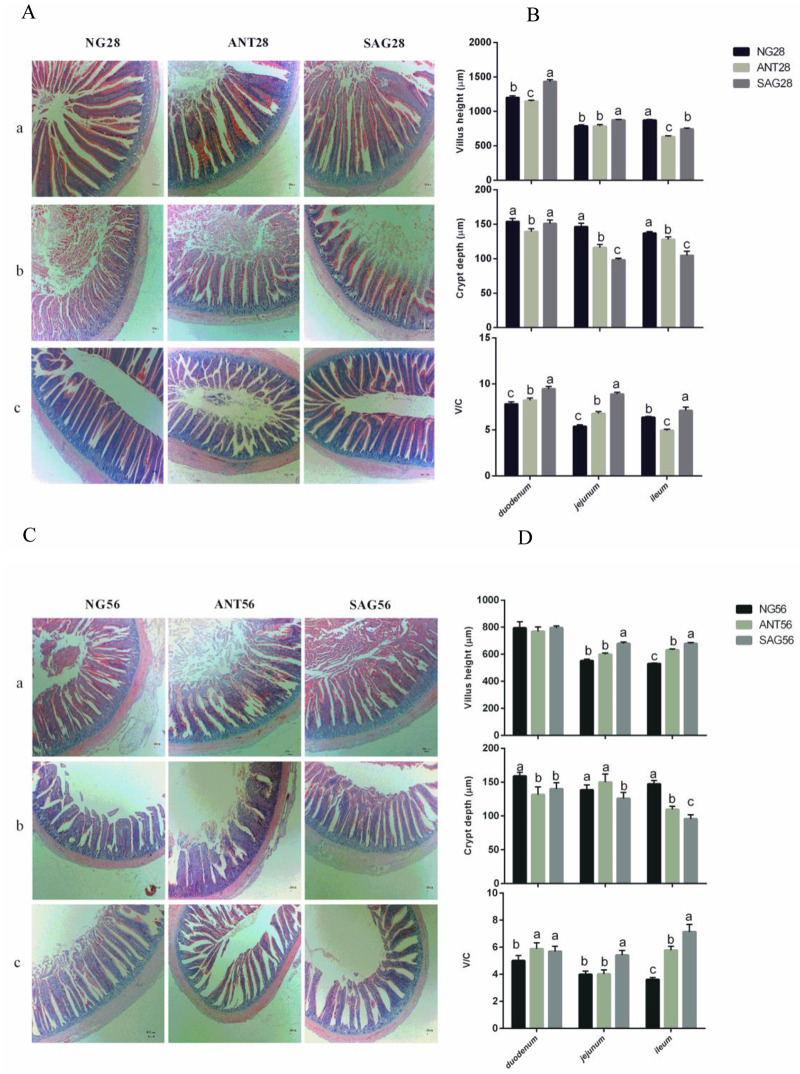
Effects of sanguinarine on intestinal mucosal morphology in yellow-feathered broilers. A: intestinal (a:Duodenum; b:Jejunum; c:Ileum) mucosal morphology in 28 days yellow-feathered broilers was observed (40 ×). B: The villi lengths, the crypt depth and the ratio of the villus length and crypt depth (V/C) were determined in intestinal in 28 days. C: intestinal (a:Duodenum; b: Jejunum; c: Ileum) mucosal morphology in 56 days yellow-feathered broilers was observed (40 ×). D: The villi lengths, the crypt depth and the ratio of the villus length and crypt depth (V/C) were determined in intestinal in 56 days. Values are means ± SE, n = 6. Different superscripts lowercase letters within each group mean different significantly (P<0.05). NG: basal diet; SAG: sanguinarine supplemented with 0.7 mg/kg of diet; ANT: basal diet group with 50mg/Kg BMD.

The changes observed in intestinal mucosal morphologies including the duodenum, jejunum, and ileum are shown in Fig 6. SAG group had highest duodenum and jejunum villus height compared with other groups (p<0.001) at 28d, and highest jejunum and ileum villus height compared to the other groups at 56d (p<0.001). NG group had highest ileum villus height compared to be other groups at 28d (p<0.001). Subsequently, the SAG group had the least intestinal crypt depth at jejunum and ileum compared with the other treatment groups during the feeding period (p<0.001). The ANT group had the least intestinal crypt depth at the duodenum compared to the other groups at 28d (p<0.001). On the other hand, the NG group had the lowest villus height/crypt depth ratio (V/C) in duodenum and jejunum compared with other groups (p<0.001) during the feeding period. ANT group had the lowest V/C in ileum compared with other groups (p<0.001) at 28d.

## Discussion

### Sanguinarine and BMD effect on chicken hindgut microbial community

As observed in the current study, the effects of sanguinarine and BMD supplementations on microbial community α and β-diversity were different but not significant in broilers. This is consistent with a previous finding that treatment with avilamycin strongly impacted microbiota composition in the ileum than in the cecum[37]. Considering that the hindgut has a much higher microbial cell density and microbial diversity, the microbiota in the hindgut is more stable and less affected by feed additives[38]. Our data showed that taxonomic profiling demonstrated that sanguinarine treatment could reduce the level of Proteobacteria. This is consistent with a previous observation that *Salmonella* Enteritidis-challened broilers with sanguinarine via drinking water reduced *Salmonella* Enteritidis count in the cecum [39]. Huang et al. found that *Macleaya cordata* extract has positive regulation of beneficial *Lactobacillus* and the negative regulation of some commensal and pathogenic bacteria constituents in chickens[40]. Similarly, the present experiment showed that birds receiving the sanguinarine diet had an abundance of *Ruminococcaceae*, *Clostridium*, and *Lachnospiraceae*, *Clostridium*, *Butyricicoccus*, *and Faecalibacterium* which are *SCFA* producers[41,42]. Dietary supplementation with sanguinarine increased the concentrations of acetate, propionate, butyrate, valerate and total SCFAs in ileal and caecal samples of early-weaned piglets [43]. These results indicated that sanguinarine may promote a more symbiotic intestinal microflora favoring the host.

### Sanguinarine effect on intestinal mucosal morphology and mucosal inflammatory response

In this study, we found that villus heights were shorter in the ileum of broilers fed with sanguinarine when 28d of age. Similarly, dietary supplementation with sanguinarine diminished villus height in the duodenum of broiler chickens[44]. Furthermore, we also found that villus heights were higher in the duodenum and jejunum of broilers fed the sanguinarine than those from the other groups during the feeding period. At 56d of age, we also found that the villus heights were higher in the ileum of broilers fed with sanguinarine than those from the other treatment. Similarly, Lee et al. demonstrated that broiler chickens fed diets supplemented with sanguinarine increased relative jejunal and ileal lengths[23]. Hence, our results indicated that dietary supplementation with sanguinarine played a beneficial role in improving the intestinal morphology of the birds. Sanguinarine was found to block tumour necrosis factor-alpha (TNF-α) induced phosphorylation and the degradation of IκBα, an inhibitory protein of nuclear factor (NF)-κB, as well as inhibiting the translocation of the p65 subunit to a nucleus [45]. In this study, the concentrations of TNF-α and IL-4 were decreased in sanguinarine group, it is similar to the reported that sanguinarine has anti-inflammatory property in vitro and in vivo, and showed a bility to reduce the secretion of TNF-α [46, 47].

### Sanguinarine promote chicken growth

The present study showed that dietary sanguinarine supplementation enhanced ADG of broiler significantly at 28d age, and this effect disappeared after 28d. Our results are consistent with the conclusion of Vieira et al, which indicated that sanguinarine supplementation improved significantly BW of broilers in the early stage[48]. Our results showed that sanguinarine supplementation significantly enhanced ADFI significantly in the periods of 1 to 28 d age, although the previous study reported that sanguinarine addition increased feed intake and decreased FCR of broilers only during 22-35d of age[23]. These contrasting effects of sanguinarine on performance might be related to the content of sanguinarine supplied, the length of the experimental period or the species tested[49–51]. Sanguinarine has a structurally related quaternary benzo[c] phenanthridine alkaloid, it exhibited an irreversibly inhibitory influence on intestinal aromatic amino acid decarboxylase[52]. Amino acid decarboxylase like aromatic amino acid decarboxylase catalyses the decarboxylation of amino acid to their subsequent biogenic amines [53]. Sanguinarine can improve the protein retention by reducing the intestinal decarboxylation of aromatic amino acids, adjust pH of the intestine, enhance feed intake by modulating effects on the Trp-serotonin pathway, thereby, promoting animal growth[52,54]. Those responses might explain why yellow-feather chickens receiving this supplement in our study showed better growth performance.

### Sanguinarine effect on serum biochemical parameters

The present study showed that added sanguinarine had a limited effect on the birds’ serum biochemical parameters which indicated no negative action of sanguinarine on the health status of birds[3,55]. In contrast to our findings, the addition of sanguinarine in the diet led to a reduced serum cholesterol content, serum albumin percentage, higher globulin percentage and gamma globulins in hens[56]. However, our results showed that dietary sanguinarine supplementation did not significantly affect serum total protein, ALB, GLB, ALT, AST, TG, TC, HDL, and LDL content. In agreement with our observations, Kosina et al. found that the inclusion of sanguinarine had no influence on serum albumin, globulin and total protein levels in pigs[51]. On the other hand, the birds that received sanguinarine had decreased serum GLU content significantly. That result was in accordance with the ADG performance, indicating that sanguinarine improved absorption and utilization of GLU, resulting in higher growth. The birds that received sanguinarine decreased serum UA and BUN levels significantly, which was in agreement with the results that dietary sanguinarine supplementation increased serum contents of amino acids, such as Gly, Ile, Lys, Met, Arg, Ala, and Thr in growing pigs[57]. Those trends were in accordance with the growth performance, suggesting that the sanguinarine enhance protein synthesis, resulting in less serum UA and BUN and higher body and muscle growth, which finally led to a higher body weight performance.

### Sanguinarine maintain the chicken gut mucosal morphology and microbiota to promote growth

The contents of Firmicutes and Firmicutes/Bacteroidetes ratio were gradually increased while the content of Bacteroidetes was decreased with increasing body mass index (BMI) in humans[58,59]. In line with this, our results showed that Firmicutes and the ratio of Firmicutes/ Bacteroidetes were positively correlated with the ADG. Sanguinarine and BMD supplementation in the diet could increase their ADG of birds, hence, the increase in Firmicutes/Bacteroidetes ratio. A possible explanation for our findings is that Firmicutes are more effective as an energy source than Bacteroidetes, thus promoting more efficient absorption of carbohydrate and subsequent weight gain[60,61]. In the present study, increased in villus height and villus height: crypt depth ratio were also observed in the small intestinal mucosa of broilers supplemented with sanguinarine. Such improved intestinal mucosal morphology may be explained by the lower numbers of proteobacteria and lower pH of the small intestine[50,62]. The decrease of pathogens is likely to alleviate the host inflammation and immune responses, and indeed, our data showed that the host cytokines including IL-4 and TNF-α were decreased. These results suggest that sanguinarine might inhibit the action of harmful bacteria in the intestinal wall and reduced the production of toxic compounds and avoid the damage to intestinal epithelial cells, thereby protecting the intestinal mucosa[31]. The change in villi and crypt depth demonstrated that sanguinarine could broaden the absorption area of the small intestine and improved the capability of digestion and absorption. Taken together, these results suggest that the growth promotion may be achieved by the enhancement of microbial community and gut mucosal morphology by sanguinarine supplementation.

## Conclusion

We have elucidated that sanguinarine supplemented at 0.7mg/kg could improve mucosal morphology and the cecal microflora of yellow broilers thereby increased their body weight gain at 28 day of age.

## Supporting information

S1 Data (XLS)

## Abbreviations

ADFI: Average daily feed intake
ADG: Average daily body weight gain
ALB: Albumin
ALT: Alanine aminotransferase
ANT: Basal diet containing BMD
AST: Aspartate aminotransferase
BMD: Bacitracin methylene disalicylate
BUN: Blood urea nitrogen
FCR: Feed/gain ratio
GLB: Globulin
GLU: Glucose
H&E: Hematoxylin and eosin
HDL: High density lipoprotein
LDL: Low density lipoprotein
NG: Basal diet
OTU: Operational taxonomic unit
PCoA: Principal coordinates analysis
SAG: Basal diet containing sanguinarine
TC: Total cholesterol
TG: Triglycerides
TNF-α: Tumor necrosis factor-a
TP: Serum total protein
UA: Uric acid
V/C: Villus height/crypt depth
